# Viewing the rare through public lenses: insights into dead calf carrying and other thanatological responses in Asian elephants using YouTube videos

**DOI:** 10.1098/rsos.211740

**Published:** 2022-05-18

**Authors:** Sanjeeta Sharma Pokharel, Nachiketha Sharma, Raman Sukumar

**Affiliations:** Centre for Ecological Sciences, Indian Institute of Science, Bengaluru, India

**Keywords:** *Elephas maximus*, carcass carrying, death reactions, rare behaviour, thanatology, YouTube

## Abstract

Documenting the behavioural repertoire of an animal species is important for understanding that species' natural history. Many behaviours such as mating, parturition and death may be observed only rarely in the wild due to the low frequency of occurrence, short duration and the species' elusiveness. Opportunistic documentation of rare behaviours is therefore valuable for deciphering the behavioural complexity in a species. In this context, digital platforms may serve as useful data sources for studying rare behaviours in animals. Using videos uploaded on YouTube, we document and construct a tentative repertoire of thanatological responses (death-related behaviours) in Asian elephants (*Elephas maximus*). The most frequently observed thanatological responses included postural changes, guarding/keeping vigil, touching, investigating the carcass, epimeletic behaviours and vocalizations. We also describe some infrequently observed behaviours, including carrying dead calves by adult females, re-assurance-like behaviours and attempts to support dying or dead conspecifics, some of which were only known anecdotally in Asian elephants. Our observations indicate the significance of open-source video data on digital platforms for gaining insights into rarely observed behaviours and support the accumulating evidence for higher cognitive abilities of Asian elephants in the context of comparative thanatology.

## 1. Introduction

Documentation of natural history traits is a key prerequisite to an adequate understanding of the behaviour of an animal species. Understanding proximate (how) and ultimate (why) causations underlying behaviours has been a concern of naturalists and behavioural ecologists for decades, even centuries [1]. Questions about species' foraging, mating, movement, predation or social interactions are often framed as how and why animals interact in specific ways with and within their environments. In addition, quantifying and interpreting a particular behaviour in a species may be further confounded by individual variations and behavioural flexibility exhibited in different physical and social environments [2–4]. Although some traits and behaviours (such as foraging, predation, movement) are relatively easy to observe and quantify, others are less so, because they occur only either infrequently or ‘rarely'.

A ‘rare' behaviour might simply be one that occurs at low frequencies relative to other behaviours or is difficult to observe directly due to unfavourable field conditions (for instance, terrain, dense vegetation, and aquatic environment limiting visibility and navigation) or animals' elusiveness. A detailed description of rarely observed behaviours (parturition, death, reciprocity, sleep) [5–9] requires the observer to be at the right place at the right time. With appropriate caution and interpretation, rare events and anecdotes can provide valuable insights into a species' life-history traits and thus lead to advances in behavioural theories [8,9–11]. For example, several important discoveries such as tool-use and innovation in chimpanzees (*Pan troglodytes*) [12], tactical deception in chacma baboons (*Papio ursinus*) [13], self-medication [14] and elaborate responses to dead conspecifics in various species [15–17] began with accumulating field notes and anecdotal reports from various study sites. Among these developments, ‘comparative thanatology', the study of animals' responses to death, has burgeoned recently, with a particular interest in the case of highly social taxa with complex cognitive abilities [18–20].

Reports of various animals' thanatological responses have been increasing in recent years (for a detailed review, see [17]). These comprise anecdotal reports on a wide range of taxa including different social mammals, contributing to the development of comparative and evolutionary perspectives concerning ‘death awareness' in animals [15,16,18,21]. Components such as inevitability (all life forms are mortal), non-functionality (dead individuals cannot perceive and/or feel), irreversibility (the dead cannot be revived) and causality (cessation of biological functions) are important in defining human-like understanding of death [15,21]. Recent research and debates in comparative thanatology have challenged the popular belief that only humans have an understanding of death, hinting at the existence of some degree of death awareness in different animal taxa [16].

Non-human animals display remarkably varied reactions when confronted with the deaths of conspecifics. Among the most frequent and common responses are direct responses such as touching, sniffing, epimeletic responses and dragging or carrying the corpse; among mammals, these can last from a few minutes to weeks or even months. ‘Secondary' reactions have also been documented, for example, guarding, vigilance, mobbing, various vocalizations (sometimes resulting in ‘cacophonous aggregations'), avoidance and aggression towards the corpse or bystanders (reviewed in [16,17]). Thanatological reactions are based on different types of signals emanating from the dead individual. In social insects, ‘death scents' or ‘necromones' elicit ‘necrophoric' or risk-aversive behaviours including avoidance, removal, or in some species, burial of the body [16,21]. Although insects show some degree of flexibility in how they respond to dead individuals, their behaviours are largely stereotypic, likely with no underlying cognitive awareness of death [21].

Some mammalian species, such as proboscideans, that live in complex societies with strong social bonds among kin and possessing higher cognitive abilities perhaps display greater interest towards their dead conspecifics. In the African savanna elephant (*Loxodonta africana*), several studies have documented strong interest in dead conspecifics (expressed by investigating, repeatedly visiting or touching the deceased, as well as self-directed reactions), irrespective of their genetic relationship with the dead individual [22–25]. They show greater inquisitive behaviour towards deceased conspecifics than heterospecifics [26]. A study on African elephants showed that the herd members reacted to playback calls of former members (a dead female that had died during the study and another female that had left the natal herd) suggesting long-term memory and vocal recognition of individuals [27].

By contrast, scientific documentation is scarce regarding how the Asian elephant (*Elephas maximus*), a species with similar societal dynamics and cognitive ability to its African counterpart, reacts to dying and dead conspecifics. Asian elephants live in matrilineal, multi-levelled fission–fusion societies [28,29]. Compared to African savanna elephants, Asian elephants live in relatively small groups with weaker and nonlinear dominance networks among social units [30], and associations between females that tend to be temporally stable across seasons and years [29]. The importance of interactions among individuals and herds in elephant societies raises the issue of survivors' psychological responses triggered by the death of a group member [31]. However, Asian elephants remain largely unstudied in terms of thanatological behaviours, especially under free-ranging conditions.

Asian elephants mainly inhabit mixed to closed forests [32] making observation of rare behaviours difficult, which at least partly explains the paucity of information on Asian elephant thanatology (but see [33]). In this context, the growth and popularity of digital and social media platforms (for example, YouTube) may provide valuable sources for documenting some rare behaviours [34,35] including thanatological reactions. Of late, records and anecdotal events obtained from public digital platforms are being increasingly used in scientific studies of various topics [36–38] including prey–predator interactions [39], human–wildlife interactions [40], and conservation and welfare-related issues of various taxa [41–44].

Therefore, this study constructed a detailed repertoire of thanatological responses of Asian elephants in captivity, semi-captivity and free-ranging conditions using opportunistic observations uploaded by the public to a digital platform, YouTube.

## 2. Methods

### 2.1. YouTube video data selection

We searched for videos on YouTube (https://www.youtube.com/) using keywords and phrases including ‘death of elephants', ‘elephant reactions to death', ‘elephant death', ‘calf elephant death’, ‘elephant responding to death', ‘Asian elephant death' and ‘dead elephants'. Google translate was used to translate into the regional languages of some Asian elephant ranging countries (for instance, Kannada, Malay, Thai). Out of 58 videos related to the above search phrases, only those showing thanatological behaviours in Asian elephants were retained (*n* = 39 videos showing 24 cases). The video search was conducted between May 2020 and June 2021. Each video was numbered, and for each, we recorded the year of publication, name of the channel and URLs, the country in which the event was filmed, status (captive, semi-captive or wild) and age/sex of dead and responding individuals (electronic supplementary material, table S1). Some videos relating to the same incident were uploaded on different channels; in these cases, the videos were all coded to extract any relevant additional information (*n* = 39 videos for 24 YouTube cases; electronic supplementary material, table S1). Additionally, two videos (of the same incident; electronic supplementary material, video S1a and S1b) from the data archive of one author (R.S.) were included as ‘non-YouTube case'. Each case was numbered consecutively from Case #1 to Case #25 (total cases = 25 including one non-YouTube case; table 1; electronic supplementary material, tables S1 and S2) and responders' sex and age-class (see note to electronic supplementary material, table S2) were coded using a combination of letters and numbers (for example, F1 referring to first adult female in Case #1; F2, F3 and F4 to three adult females in Case #2). Behaviours were coded by the author S.S.P. The YouTube videos were used only for this research, adhering to the fair use and Creative Commons license (videos uploaded in the ‘public domain') policies of YouTube, and all content uploaders are duly acknowledged (figure 3; electronic supplementary material, figures S1–S3 and table S1).

**Table 1.** Detailed thanatological reactions based on 24 YouTube cases (*n* = 39) and 1 non-YouTube case (*n* = 2; Case #25) on Asian elephants. For more details, refer to electronic supplementary material, tables S1 and S2.

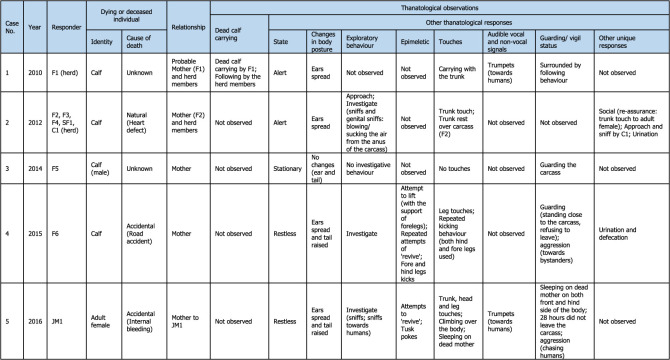
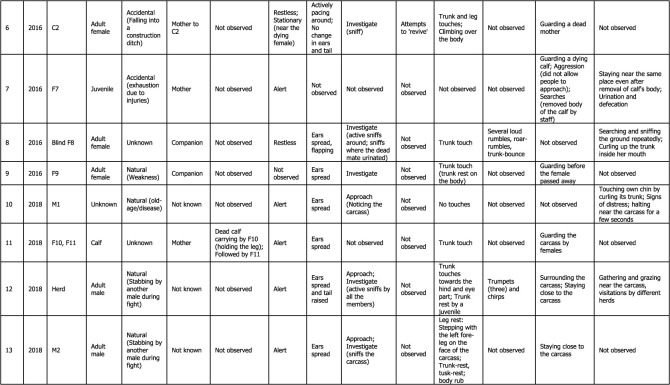
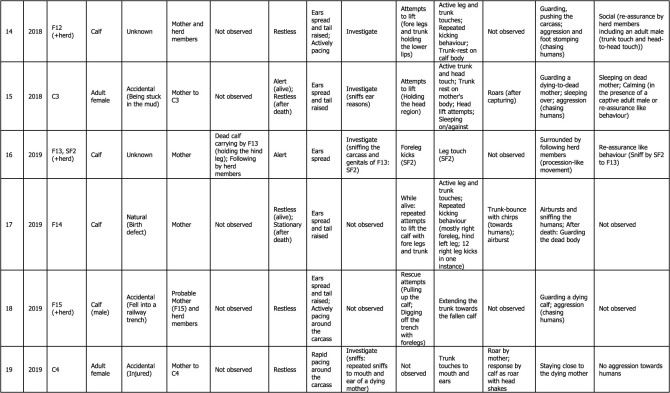
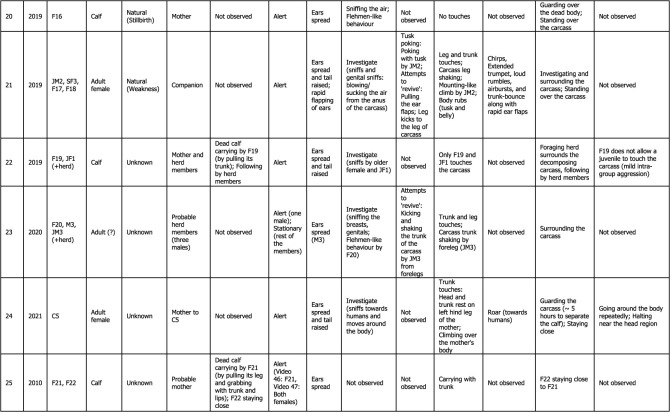

### 2.2. Assessment of thanatological behaviours

Visible behaviours directed towards the dying or dead individuals were noted as ‘visual thanatological responses'. For analysis, videos were viewed while playing between 0.25 and 0.5 times slower than normal, and records were made concerning: (a) the year of the incident, (b) responder (identity: sex and age class by relative height method [45]; status: wild, captive or semi-captive), (c) dying or dead individual (identity, status, age if known, cause of death either natural, accidental or unknown; based on the description provided), (d) carcass decomposition status (dying or freshly dead, fresh, decomposing, advanced decomposing), (e) relationship to the responder (mother, calf, herd member, companion (if captive or semi-captive), unrelated or unknown), (f) number of elephants present in the video (probable herd size including responders and dying or deceased individual), (g) clip duration (excluding frames unrelated to thanatological behaviours), (h) visual thanatological responses and (i) types of human intervention (table 1; electronic supplementary material, table S2). The presence of humans or types of human intervention were categorized into four broad classes: (i) ‘no disturbance' (no obvious direct human involvement other than the presence of the videographer and any companions), (ii) ‘controlled' interventions (responder's behaviour may be influenced by factors such as fences or the presence of *mahouts* (elephant handlers)), (iii) ‘rescue' attempts (when responder reactions may have been influenced by attempts to rescue or treat the dying individuals) and (iv) ‘disturbance' (e.g. the presence of a wildlife safari or villagers, recovering and transporting or cremating the deceased, vehicular traffic; electronic supplementary material, table S2).

**Figure 1.**
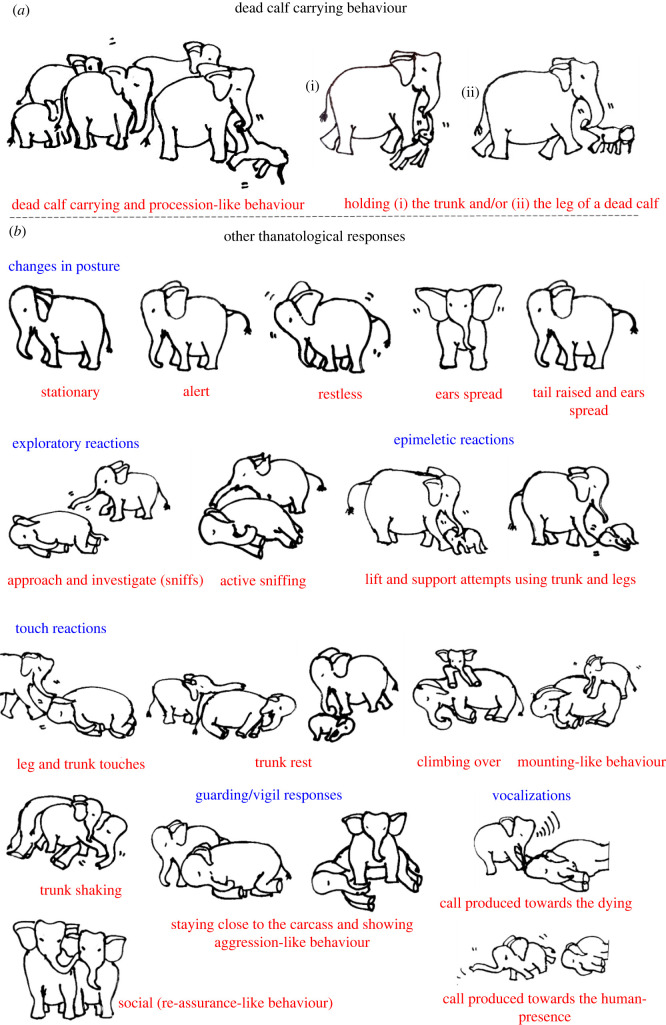
Illustrations showing (*a*) dead calf carrying behaviour and (*b*) some of the other visual (to the assessors) thanatological responses (change in posture, exploratory, epimeletic, touches, guarding/vigil, vocalizations and social behaviours). (Illustrations by S.S.P.)

Visual thanatological behaviours were divided into two broad categories for ease of analysis and interpretation, namely, ‘carrying' a dead calf (including holding), and ‘other', which included: (a) postural changes (further categorized as ‘stationary’: standing still; ‘alert': ears spread and tail raised; and ‘restless': ears spread, tail raised while actively pacing around the dying or dead individual), (b) exploratory behaviours (including visiting, approaching, actively sniffing or investigating and flehmen-like behaviours towards the individual), (c) epimeletic behaviours (including attempts to support or lift the individual with trunk and/or leg, or by kicking, shaking, pulling or poking with tusks or tushes), (d) touch (simple contact with trunk, head, leg or body; included trunk touch or rest, leg touch, trunk or leg shake of the carcass, climb or sleep on the individual, or mount-like attempts, kicks, pokes or nudges), (e) audible vocal (trumpets, rumbles, chirps, roars and combination of these calls) and non-vocal sounds (air-bursts, trunk bounce, ear-flaps) [46,47]; although difficult to do with certainty, based on the video evidence calls were classified as either conspecific- or human-directed, (f) guarding/vigil (staying close to or surrounding the carcass, refusing to leave, displaying ‘protective' behaviour and ‘aggression-like' behaviour towards humans such as charging) and (g) any other responses (such as sleeping against or on an injured mother (died later), and social (re-assurance-like behaviours); figure 1 and table 1; electronic supplementary material, figures S1–S3). Visual thanatological reactions were qualitatively categorized based on the content of the videos and were accordingly labelled. For example, ‘repetitive kicking behaviour': if a responder kicked repeatedly; a ‘trunk touch': if a responder was observed touching with its trunk; and a ‘dead calf carrying': if a responder was observed carrying a dead calf (figure 1; electronic supplementary material, figures S1–S3).

### 2.3. Data analyses

Descriptive statistics were used, namely, frequency and percentages of occurrence. The number of videos in which a behavioural reaction was present was referred to as the total frequency of such behaviour. For example, one ‘trunk touch' for one video was counted if a responder touched the dying or dead individual at least once. All videos with ‘trunk touches' were then summed and plotted to represent the total frequency of ‘trunk touch' observed across all cases (figure 4*b*(ii) and table 1). However, the frequency of ‘trunk touches’ within each video was not calculated. Any above-mentioned behavioural categories not present in a video were coded as ‘not observed' (table 1). The relative frequency (percentages of occurrence) of each visual thanatological response was calculated by dividing the number of cases showing a thanatological response by a total number of cases (*n* = 25) and was expressed as a percentage (figure 4*a*).

## 3. Results

**Figure 2.**
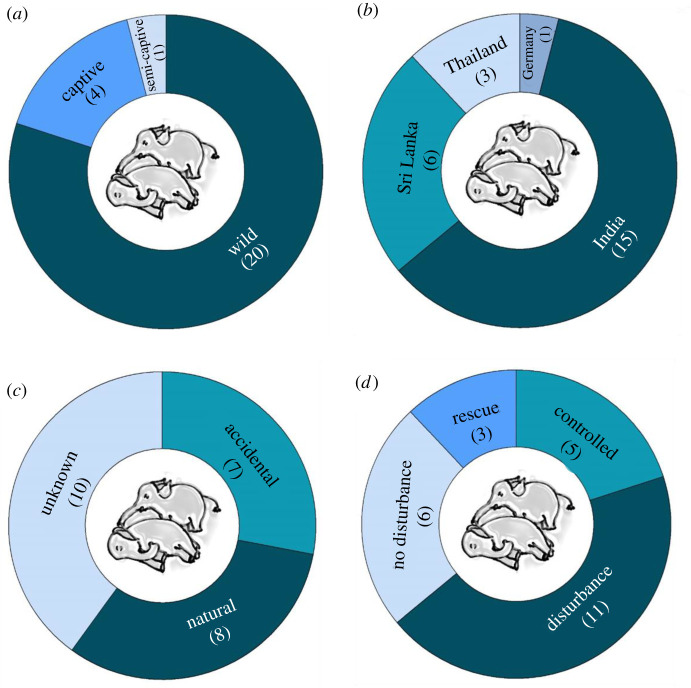
Doughnut charts representing total recorded thanatological cases (presented as numbers) in terms of (*a*) animal status, (*b*) country where the cases were reported from, (*c*) the cause of deaths and (*d*) presence of humans.

A total of 24 YouTube cases (*n* = 39 videos uploaded to different YouTube channels) and 1 non-YouTube case (*n* = 2 videos of the same case) were examined (electronic supplementary material, table S1). The cases ranged between years 2010 and 2021, with most videos uploaded in 2019 (*n* = 7), followed by 2018 (*n* = 6) and 2016 (*n* = 5; electronic supplementary material, tables S1 and S2). Of the 25 cases, 80% concerned wild (i.e. free-ranging) elephants, with 16% and 4% concerning captive and semi-captive elephants, respectively (figure 2*a*). Most cases occurred in India (*n* = 15), followed by Sri Lanka (*n* = 6), Thailand (*n* = 3) and Germany (*n* = 1; figure 2*b*; electronic supplementary material, table S1). One non-YouTube case involved wild elephants in India (in the year 2010). The length of video clips containing thanatological reactions ranged from 19 to 360 s, with an average of 132.6 s (electronic supplementary material, table S2). In these videos, responders included a total of 22 adult females, three adult males, three sub-adult females, four juveniles (one female and three males) and five calves, from seven different herds. Dying or dead elephants included adult females (*n* = 8), adult males (*n* = 2), juveniles (*n* = 1; sex unknown), calves (*n* = 12; sex unknown) and age/sex unknown (*n* = 2) (table 1; electronic supplementary material, table S2).

### 3.1. Cause of deaths

The cause of death was known or reported for 60% of cases (figure 2*c*). Seven cases occurred in accidental circumstances (such as road accidents, getting stuck in the mud, falling into a ditch or railway trench, exhaustion and internal bleeding due to injury). Eight cases were categorized as natural, due to old age, pathological cases, fighting, birth defect and stillbirth. The carcasses were at different stages of decomposition: 13 were fresh, seven were dying to fresh death, one was decomposing, one decomposed and two were in an advanced state of decomposition (electronic supplementary material, table S2). In one case, the state of the carcass (of a calf) was not clear (electronic supplementary material, table S2).

### 3.2. Presence of humans

Of the 24 YouTube cases, six were categorized as ‘no disturbance', five included ‘controlled intervention', three included ‘rescue' attempts and 11 involved ‘disturbance' or direct human intervention (figure 2*d*; electronic supplementary material, table S2). One non-YouTube video had no human-induced disturbance other than the presence of a field researcher.

### 3.3. Visual thanatological reactions

#### 3.3.1. Dead calf carrying

**Figure 3.**
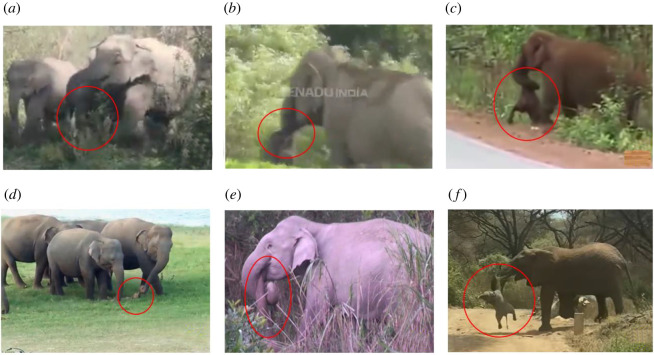
Dead calf carrying by adult female wild Asian elephants (*n* = 5 cases out of 25 total cases) and African elephant. These still images were retrieved from (*a*) Video 1 (channel: monismukhtar1), (*b*) Video 21 (channel: ETV Bharat English; Case #11), (*c*) Video 28 (channel: Buzz news; Case #16), (*d*) Video 35 (channel: Lanka Wild Safari; Case #22) and (*e*) Video 40 (non-YouTube data: from co-author R.S., data archive; Case #25; refer to electronic supplementary material, table S1, for more details). For comparative documentation, we provide a still image (*f*) from a 44 s long video of an African female elephant holding a dead calf by her tusk and trunk at the Lake Manyara National Park, Tanzania (clip source: Fay Amon, 29 August 2021; Facebook; https://fb.watch/aMDA94fK_z/).

Out of 12 total cases involving dead calves reported in this study, dead calf carrying was observed in adult female responders in four YouTube and one non-YouTube case (figure 3 and table 1; electronic supplementary material, video S1a and S1b). In these reactions, the adult female carrying the dead calf was usually not alone but accompanied or surrounded by other members of the herd (figure 3). Responders held the dead calf either by grabbing the calf's leg or trunk with its own trunk and lips (figure 1). Occasionally, the responder stopped carrying, and left the carcass on the ground (usually during feeding) before lifting it again to carry when moving away. Such carcasses were either decomposing or in an advanced decomposed state (electronic supplementary material, table S2). Dead calf carrying was not observed for dying to freshly dead calves (*n* = 7). In one case (Case #22), an adult female (F19) displayed aggression towards a juvenile (JF1), not allowing it to approach the carcass (table 1).

#### 3.3.2. Other visual thanatological responses

##### 3.3.2.1. Changes in body form or posture

**Figure 4.**
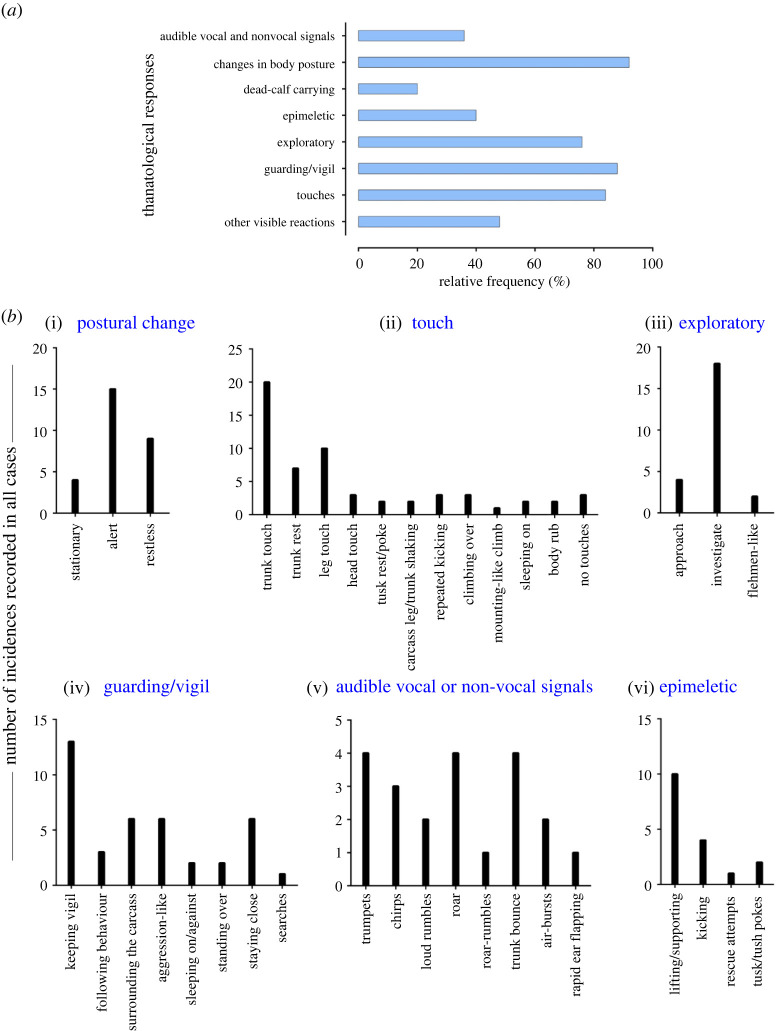
Frequency of visible thanatological responses. (*a*) Separated bar graph represents the relative frequency (%) of thanatological reactions observed in all 25 cases. (*b*) Bar plots represent some of the visual thanatological responses including: (i) changes in body posture, (ii) touch, (iii) exploratory, (iv) guarding/vigil, (v) audible vocal and non-vocal signals and (vi) epimeletic behaviours. The *x*-axes in (*b*) represent different types of responses and the *y*-axes (different scales for each plot) represent the frequency of occurrences of these reactions across all cases.

Most of the reported cases showed some forms of body posture changes (*n* = 23; figure 1 and figure 4*a*). Most responders were alert (*n* = 15) followed by being restless (*n* = 9) and stationary (*n* = 4; figure 4*b*(i) and table 1). No change in body form or posture was observed in one case. In two cases, the responder (C2, F14) was initially restless and stationary after the moment of death of the individual (Cases #6 and #17; table 1). In another case, the responder (C3) was alert before and restless after the death of its mother (Case #15; table 1). In Case #23, one of the responders, M3, showed alertness (as JM3 actively explored the carcass), while other herd members remained stationary around the carcass (table 1).

##### 3.3.2.2. Exploratory responses

There were 19 cases where exploratory responses were observed (figure 4*a*). Responders were observed investigating (*n* = 18), approaching (*n* = 4) and exhibiting flehmen-like behaviour (*n* = 2) towards the dying and dead individuals (figure 4*b*(iii) and table 1; electronic supplementary material, figure S1). One case had no investigatory behaviour and seven cases had no exploratory responses (table 1). Elephants investigated primarily by sniffing the body, breasts and genitals (blowing-in-and-sucking-the-air-like behaviours especially in the genital area, Case #2), repeatedly sniffing the mouth and ears, sniffing and moving around the carcass, sniffing the air with a raised trunk, sniffing in the direction of humans, pointing the trunk towards the deceased, sniffing the ground where the dead individual had urinated and with flehmen-like behaviour (figure 1 and table 1; electronic supplementary material, figure S1).

##### 3.3.2.3. Epimeletic responses

Epimeletic or ‘supporting’ reactions were observed in 10 out of 25 recordings, and consisted most frequently of attempts to lift, support or pull (*n* = 10; even included shaking of trunk and leg of carcasses; figure 4*b*(vi) and table 1). Kicking in an attempt to revive (*n* = 4), gently nudging with tusks/tush (*n* = 2) and trying to rescue (*n* = 1) the dying or deceased individuals were observed in these cases (figure 4*b*(vi) and table 1; electronic supplementary material, figure S3). Active use of fore and hind legs, holding the head, and pulling up the trunk, ears or lips were observed (electronic supplementary material, figures S1 and S3; table 1). Juvenile elephants used their tusks to gently nudge a dead body in two instances (Cases #5 and #21; table 1). On two different occasions, two juvenile males shook the trunk and the leg of a carcass with their forelegs (Cases #21 and #23; table 1; electronic supplementary material, table S1). Pulling up a calf that fell into a trench was among the few attempts by an adult female and herd members to succour a calf that died later (Case #18; table 1; electronic supplementary material, table S1).

##### 3.3.2.4. Touch responses

‘Touching' was the most common thanatological reaction, with 21 cases showing some forms of ‘touch' behaviour (figure 4*a* and table 1; electronic supplementary material, figures S1–S3). The most frequent were trunk touches (33.3%), leg touches (16.7%), trunk rests (11.7%), head touches (5.0%), repeated kicking (5.0%), climbing over the carcass (5.0%), tusk rests and pokes (3.3%), sleeping on or against (3.3%) and body rubs (3.3%; figure 4*b*(ii) and table 1; electronic supplementary material, figures S1 and S2). In two cases, juveniles and calves, in particular, were observed using their legs to shake the trunk or a leg of the carcass. A mounting-like reaction was shown by a juvenile male (JM2) in Case #21 (electronic supplementary material, figure S2). An adult male (M2) was observed resting its foreleg on the carcass's head (Case #13; electronic supplementary material, figure S1). Touches with the trunk (predominantly touching the eyes, mouth, ears, head regions) and both fore and hind legs were frequently observed in all responders. Responders showed no touch reactions in only three cases, engaging in guarding/vigil and approaching the carcass instead (table 1). Repeated kicking behaviour was shown by mothers (F6, F12 and F14 in Cases #4, #14 and #17, respectively), predominantly using their right foreleg and also their hind legs, towards their dying and dead calves (electronic supplementary material, figure S3; table 1). In one instance, F14 (Case #17) was observed continuously kicking her dying calf with her right foreleg nearly 12 times (table 1).

##### 3.3.2.5. Audible vocal and non-vocal responses

Audible vocalizations and non-vocal responses were evident in nine cases (the remaining 16 cases either had no vocal responses or were masked by the commentary or music added to the video; figure 4*a*). These auditory signals were directed either towards the dying or dead individuals (*n* = 21), or towards humans (*n* = 5). Vocal signals included roars (*n* = 4), trumpets (*n* = 4), chirps (*n* = 3), loud rumbles (*n* = 2) and roar-rumbles (*n* = 1), while non-vocal signals included trunk-bounces (*n* = 4), air-bursts (*n* = 2) and rapid ear flaps (*n* = 1; figure 4*b*(v)). Auditory signals that were human-directed included trumpets (*n* = 2), roars (*n* = 1), chirps (*n* = 1) and trunk-bounces (*n* = 1; table 1). Most roars were produced by calves and by a blind female (table 1). Extended trumpets along with chirps, loud rumbles, air-bursts, trunk-bounce and rapid ear flaps occurred in Case #21, produced by four responders including a juvenile male (table 1; electronic supplementary material, table S1).

##### 3.3.2.6. Guarding/vigil responses

Guarding or keeping vigil over dying or dead individuals was frequently observed (*n* = 22 cases; figure 4*a*). Keeping vigil was observed 13 times; other guarding/vigil responses included human-directed aggression (*n* = 6), surrounding the carcass (*n* = 6), staying close (*n* = 6), following an individual who carried a dead carcass (*n* = 3), remaining by the dying or dead individual and sleeping nearby (*n* = 2), standing over the carcass (*n* = 2), and one instance actively searching for a carcass that had been removed (the responder was blind; figure 4*b*(iv) and table 1; electronic supplementary material, figures S1–S3). Guarding or staying close and vigilant were seen in most responders who sometimes refused to leave even during rescue attempts (based on YouTube channels' descriptions, it took between 5 and 28 h to separate the responders from the carcass; table 1). In six cases, responders directed aggression-like behaviours (charging, trunk-bouncing, air-bursts or vocalizing) towards humans to interfere with their attempts to approaching the dying or dead conspecific (table 1).

##### 3.3.2.7. Other unique thanatological responses

A few uncommon reactions were seen in responders, in 12 cases (table 1 and figure 4*a*). These included social response (re-assurance-like behaviours such as touching each other with trunks, head-to-head touching and sniffing, or displaying calming-like reactions), frequent urination and defecation, staying at the same spot even after removal of the carcass, searching for the carcass, trunk-curling, touching own lower jaw and holding own trunk between lips, visitation by larger herds, grazing near the carcass, sleeping near a dead mother, the absence of human-directed aggression, and moving around the carcass and stopping near the head region (table 1).

## 4. Discussion

The YouTube videos that we examined yielded new information on the thanatological behaviour of Asian elephants. Despite their short duration and lack of adherence to conventional scientific procedures for collecting behavioural data, these videos allowed us to identify several thanatological responses in Asian elephants. These thanatological behaviours included carrying dead calves, touching dying or dead individuals, postural changes, guarding/vigil responses, epimeletic and exploratory behaviours, and audible vocal and non-vocal signals. However, we cannot exclude the possibility that human presence might have influenced some of the elephants' reactions. Collectively, these observations indicate that Asian elephants show interest in dying and dead conspecifics and the motivations behind thanatological responses in elephants (as in other species) need to be further explored.

### 4.1. Dead calf carrying behaviour

We observed five instances of Asian elephants carrying dead calves of unknown sex, and at various stages of decomposition (figure 3 and table 1). In the absence of confirmed evidence on relatedness, we may only speculate that the carcasses were carried by their mothers. Similar behaviour has also been observed in African savanna elephants, who were known to carry pieces of jaws, rib bones and even full-grown tusks (of dead adults), from short to long distances [16,22,25,48,49]. Carrying and interacting with fragments of carcasses are yet to be observed in Asian elephants.

Dead infant-carrying is one of the most frequently observed thanatological behaviours in primates [17,49,50], and it has also been documented in cetaceans [51,52]. The behaviour's precise underlying mechanism (both proximate and ultimate causations) is not fully understood. It remains a sparsely documented behaviour in elephants, with little clarity on its causation, ontogeny, functions and evolutionary history. Based on proposed hypotheses for similar behaviour among primates [53] and cetaceans [51,52] we can expect elephants' motivation to carry corpses may vary with the context. For example, in primates, corpse-carrying is more likely when an infant dies of illness than trauma such as infanticide [50]. Moreover, corpse-carrying is energetically costly, as moving with the corpse may hinder normal activity (e.g. foraging, mobility) [17]. Even though a small calf weighs only 3–5% of the body weight of an adult female elephant [54], carrying a dead calf by the trunk for prolonged periods would certainly hamper mobility, foraging and other uses of the trunk.

#### 4.1.1. Proximate causation of dead calf carrying

The mechanistic (how) or proximate causation of the dead calf carrying behaviour might be explained from an anatomical perspective. Morphological features such as prehensile limbs in primates may facilitate carrying a corpse while on the move. A highly dexterous, prehensile trunk (along with tusks mainly in female African elephants; figure 3*f*) perhaps enables an elephant to carry a carcass [49] without much impediment to movement. Carrying corpses in an ‘unusual' manner (primates: draped around the neck, holding one limb; cetaceans: using the mouth and dorsal fins; hippopotamidae: using the mouth; and elephants (as observed in this study): using the mouth, dragging by trunk) unlike the manner they were carried or cared for when these individuals were alive, might in itself hint at an existence of awareness that the individuals can no longer move or function on their own [17,55,56].

The duration of dead infant-carrying varies from a few hours to several weeks both within and across taxa. As observed in primates, this may depend on multiple factors including the age of the dead infant (younger infants are carried for longer) [50,57] and environmental including climatological factors [58]. For example, carrying may last longer in a terrestrial than an aquatic habitat due to faster decomposition in the latter [16,55]. Similarly, carrying duration may be extended under cold and dry conditions (due to slower decomposition) than in warm and wet conditions [59]. There is no documentation on dead calf carrying duration in elephants. Based on the states of decomposition of the carcasses in the uploaded videos, the duration of the behaviour was estimated to be a few days to weeks (figure 3); however, systematic recordings of such events are needed to gain better understandings into the short- and long-term effects of carcass carrying, at individual and group levels.

The deaths of calves can significantly alter the behaviour and personality of surviving herd members [31]. Therefore, another important question is whether dead calf carrying is mediated by the carrier's altered physiological state. Neuromodulators such as oxytocin, vasopressin and prolactin, which mediate the mother–infant relationship and stimulate affiliative interactions, might influence post-mortem carrying behaviour in females [49,53,60–62]. A captive female Japanese macaque carrying her dead infant had lower glucocorticoid levels compared to females rearing live infants, suggesting that dead infant-carrying could be a probable coping mechanism to reduce ‘stress' [63]. Though it was observed that glucocorticoid levels in adult female Asian elephants correlated positively with the number of calves in a herd (perhaps due to increased predation risks to calves, and social and nutritional stress) [64], whether carrying a dead calf has any physiological influence on the carrier remains to be clarified. Thus, longitudinal hormonal profiles of the carrier would help to provide deeper insights into the links between physiology and dead calf carrying behaviour in elephants.

#### 4.1.2. Ultimate causation of dead calf carrying

Based on similarities in terms of mother–infant bonding, the ultimate causation for dead calf carrying in extant proboscideans shows some parallels with primates. From the pertinent videos (all five cases), it is apparent that the carrying individuals were adult females or potential mothers. In elephant societies, the mother–calf pair is the fundamental unit; maternal investment, care and protection of the calf continues until the calf becomes old and strong enough to survive on its own. This prolonged period of proximity and contact fosters the development of the calf's social interactions and a strong bond with the mother and other family members [32]. As elephants are precocial species, maternal carrying of a live calf is not an innate behaviour (though a case of a mother carrying her weak calf using her tusks and trunk was reported in African elephants [65]). Therefore, even after the death, along with maternal instincts elephant mothers, like primates, may receive infantile cues from the carcass (which resembles a living calf in terms of colour, size and smell, at least for a short time) [17,55] motivating them to interact with their unresponsive calves. Our observations showed relatively older female elephants carrying a dead calf, indicating the potential influence of the mother's experiences or parity, unlike as observed in primates [50]. Thus, the maternal instinct, experiences or mother–calf bonding could be strong motivating factors behind the carrying of a dead calf in elephants. Furthermore, carrying of a dead calf by an elephant may indicate either lack of understanding of death cues and hence perceiving it as an unresponsive calf, or perhaps might imply a ‘grief-like’ response (responses generated when animals associate with each other beyond survival-oriented purposes (mating, foraging, migration) altering their normal behaviours in the context of death) [66].

### 4.2. General thanatological observations

The most common thanatological behaviour by elephants in this study was the touch response. Responders repeatedly touched the body parts of the deceased using the trunk and the legs. Other touch responses included trunk rest, tapping and shaking the trunk or leg of the deceased, and climbing on and mounting-like postures on the dead body (by young individuals). Touching the corpse was also the most frequently observed behaviour in African elephants [25]. These observations highlight the importance of tactile communication in elephant societies. Through touch, elephants may obtain cues on the well-being of other individuals [67]. Touch reactions such as repeated kicking of a calf carcass may also occur in non-thanatological contexts; for example, after giving birth one adult female repeatedly kicked and twisted the trunk of an asphyxiated new-born calf in an apparent attempt to invigorate it (https://www.youtube.com/watch?v=97CRwd_U2FU&ab_channel=MasonElephantPark%26Lodge; 20 September 2009), and may have a similar function in a death-related context. Additionally, during stressful events, Asian elephants often use their trunk to touch each other's heads, mouths and genitals as an act of reassurance [68]. Although uncommon in this study, similar behaviours were possibly used as consoling gestures in response to others' emotional state of ‘grieving'.

Investigating the corpse and approaching an ailing individual were frequently observed in the videos. Exploratory behaviours may help individuals to obtain multi-sensory information about potential danger, for example, from predation or pathogens [16,25]. Herd members near the dead individuals displayed changes in body postures including spreading of the ears (sometimes with flapping) and raising the tail in alert, usually while in an agitated state. Though similar behaviours have been recorded during thanatological observations in African elephants [25], from our video observations, we could not ascertain whether postural changes were reactions to the corpse or the presence of humans.

Epimeletic responses such as supporting or attempting to lift the dying or dead individual have been reported in various social mammals including primates [17,55] and cetaceans [51,52,69]. Elephants frequently use their legs, trunk and tusks to lift fallen conspecifics [25,33]. A kin-bonded social structure in elephants may act as a key driving force for the herd members to continue to care for and assist the ailing and dead conspecifics. Another form of thanatological behaviour is guarding; this response might include aggression towards intruders [33] and even towards members of the same group [17]. An unwillingness to leave the corpse might protect it from predators or scavengers. Eventually, the abandonment of the corpse might be evidence of some comprehension of the state of the dead individual, such as the permanence of the inertness.

The videos allowed us to identify different types of audible vocalizations and non-vocal signals produced by the elephants. These acoustic signals appeared to be directed towards both humans and conspecifics. The most frequent vocalizations were trumpets, roars and chirps. Loud rumbles and combination calls (roar-rumbles) were also detected, albeit rarely. Asian elephants display a wide vocal repertoire, with calls produced in various contexts [46,47,70] that are associated with modulations of the acoustic properties of the calls [71]. Trumpets, chirps, roars, rumbles and combination calls, produced during social interactions usually express threats or distress and serve as contact or alarm calls [46,47]. Previously in Asian elephants, trumpets were recorded in thanatological contexts [33], but functions of elephant vocalizations produced in the context of death are yet to be explored. This is also true for low-frequency vocalizations that were not recorded on the videos. Similarly, the association between non-vocal signals or a potential gesture of restlessness such as trunk-bounce, air-burst (forceful expulsion of air through trunk) and rapid ear flapping with the overall psychological and emotional state of the surviving herd members requires further study.

Our observations have revealed further similarities between thanatological behaviours between Asian and African elephants. These species' thanatological responses show some remarkable resemblances with certain species of primates and cetaceans having strong social bonding and large brains with advanced cognitive capacities [19,20]. We hope that future research will explore plausible hypotheses pertaining to the evolution of thanatological responses in proboscideans, considering the species' social dynamics, ecology and sensory-cognitive capabilities. Cross-species comparisons of elephants would help to construct an accurate picture of the evolutionary origins and maintenance of thanatological behaviours in extant proboscideans.

### 4.3. YouTube videos as a supplementary source for the study of animal behaviour

The unprecedented rate of digital data accumulation in recent times has opened up new opportunities to gain information about the natural world. Jarić *et al.* [34] introduced a new approach termed ‘iEcology’, proposing that digital data available on social media sites can be an important supplement for studying multiple topics in animal behavioural ecology from temporal and spatial perspectives. The repository of videos uploaded onto one digital platform, YouTube, provides a useful source of information for surveying an array of topics related to wildlife ecology and conservation, including animal behaviour [36,38], threats related to biodiversity [40,72,73], attitudes towards conservation [74,75] and hypothesis testing [76]. In addition, these videos promote public engagement in science. The present study provides further evidence that videos from social media sites can indeed be useful for examining animal behaviours that are only rarely observed directly. Although this study contributes new information about the thanatological behaviours of Asian elephants, we acknowledge that the secondary source (social media) of data is not problem-free for scientific analyses. As the videos came from various channels and content creators, the observations made here could represent only an unknown fraction of the entire thanatological repertoire of elephants. For instance, observations related to dead calf carrying would be influenced by natural factors such as depredation or scavenging of a carcass, lack of longitudinal observations (from the time of death of the calf) and other recording biases. Therefore, when possible, systematic recording of death-related behaviours in elephants would greatly contribute to the field of comparative thanatology.

## Data Availability

We have used publicly uploaded YouTube videos as primary data for our study. We have coded the selected videos to construct the tentative repertoire of thanatological responses in Asian elephants. The outcome from qualitative analyses is presented as figures (figures 1–4; also includes still images with credits provided in the figure legend and illustrations by S.S.P.) and the categorized behaviours are presented as a table (table 1). We have provided the raw files as electronic supplementary material (as word files and videos) as supplementary figures, videos and tables. The datasets and other supporting information related to this article have been uploaded as part of the electronic supplementary material [77].
